# Enhanced uncertainty sampling with category information for improved active learning

**DOI:** 10.1371/journal.pone.0327694

**Published:** 2025-07-07

**Authors:** Xiaochuan Wang, Bo Zhang, Fei Wang, Tao Bao, Zhiqing Lu, Jiawei Bao

**Affiliations:** 1 China Ship Scientific Research Center, Wuxi, China; 2 Taihu Laboratory of Deepsea Technology Science, Wuxi, China; Shanghai Maritime University, CHINA

## Abstract

Traditional uncertainty sampling methods in active learning often neglect category information, leading to imbalanced sample selection in multi-class computer vision tasks. Our approach integrates category information with uncertainty sampling through a novel active learning framework to address this limitation. Our method employs a pre-trained VGG16 architecture and cosine similarity metrics to efficiently extract category features without requiring additional model training. The framework combines these features with traditional uncertainty measures to ensure balanced sampling across classes while maintaining computational efficiency. Extensive experiments across both object detection and image classification tasks validate our method’s effectiveness. For object detection, our approach achieves competitive mAP scores while ensuring balanced category representation. For image classification, our method achieves accuracy comparable to state-of-the-art approaches while reducing computational overhead by up to 80%. The results validate our approach’s ability to balance sampling efficiency with dataset representativeness across different computer vision tasks. This work offers a practical, efficient solution for large-scale data annotation in domains with limited labeled data and diverse class distributions.

## 1. Introduction

In the rapidly evolving landscape of modern information technology and next-generation artificial intelligence, traditional ship technologies are encountering both new opportunities and challenges. Object detection technology is one of the essential means for achieving intelligence and unmanned capabilities in the field of ships. However, this technology often requires a substantial amount of annotated and comprehensive image or video data. Handling large datasets with high annotation precision requirements can incur significant time and manpower costs. Active learning methods can selectively identify high-value data samples from unlabeled datasets, reducing the annotation workload and serving as a primary approach to address the challenges.

Active learning can be formally categorized into two types: data stream-based sampling [1] and pool-based sampling [2–4]. In the intelligent ship domain, there is a vast amount of unlabeled data collected through sensors such as optical, sonar, and radar. Therefore, research on pool-based active learning is crucial and urgent. The key to active learning lies in the design and selection of sampling strategies. Existing methods can be primarily categorized into uncertainty-based active learning methods and deep active learning methods. Among uncertainty sampling strategies [5–8], there are strategies such as lowest confidence sampling [9], margin sampling [10], and image entropy sampling [11,12]. In [13], the approach involves utilizing an existing historical model to predict the posterior probability of unlabeled samples and selecting the most uncertain ones. This method relies on a pre-trained historical model and cannot effectively measure the instability of an entirely new sample pool. In [14], a two-layered distinct selection strategy is employed, considering the uncertainty, distinctiveness, representativeness, and distribution information of samples. However, the distribution information cannot accurately express the category information of the samples.

Due to its enhanced feature extraction capabilities in handling high-dimensional complex data such as images and videos, the integration of deep learning and active learning [15,16] has become a mainstream approach. In [17], a semi-automatic labeling system based on deep active learning is proposed, applying active learning in the field of medical image analysis. In [18], a deep active learning method designed for object detection is proposed. It utilizes a mixture density network to estimate the probability distribution of outputs from each localization and classification head. [19] introduced an approach for optimizing active learning (AL) algorithm selection through differentiable query strategy search methodology. However, this approach’s computational overhead and optimization efficacy are potentially constrained by both the cardinality of candidate strategies and the inherent complexity of the data distribution. However, these methods, when confronted with new problems or data pools, require the retraining of deep neural networks, incurring substantial time and computational costs.

Based on the preceding analysis, the fundamental limitation of traditional uncertainty sampling methods lies in their singular evaluation mechanism that solely considers sample uncertainty while neglecting class-specific information. In complex multi-class scenarios, such methods tend to generate significant class imbalances within datasets: high-frequency or high-complexity classes (such as large dock facilities and vessels) become overrepresented in the sample pool, while low-frequency classes (such as lighthouses and navigational markers) suffer from insufficient representation. This distributional imbalance severely constrains model performance, resulting in significantly diminished predictive capability for underrepresented classes and ultimately affecting overall accuracy. Moreover, existing deep learning-based sampling strategies typically require the pre-construction of training sets for model pre-training, which substantially increases computational load and time expenditure.

To overcome these limitations, this research proposes an enhanced active learning framework that innovatively integrates class information with traditional uncertainty sampling methods. Through the incorporation of a pre-trained VGG16 [20] architecture and cosine similarity metrics, this framework achieves efficient feature extraction and classification, assigning class identifiers to each candidate sample prior to sampling. This integration mechanism ensures balanced sampling across classes, significantly enhancing dataset representativeness while maintaining high computational efficiency. Compared to existing deep learning-based active learning methods, our proposed approach effectively mitigates class imbalance issues while reducing computational complexity, achieving efficient active learning across the entire sample pool. Accordingly, the primary academic contributions of this research can be summarized in the following three aspects:

(1) We propose an active learning method based on uncertainty sampling that integrates category information.
(2) Our approach utilizes a pre-trained VGG16 and cosine similarity algorithm to extract category information from the deep features of images, mitigating the long-tail effect in the sampled dataset.
(3) Compared to deep learning methods, our approach significantly reduces computational requirements and time costs. Consistent with traditional uncertainty sampling strategies, it achieves active learning across the entire sample pool.

The remainder of this paper is organized as follows: Section 2 introduces three traditional uncertainty sampling strategies. Section 3 discusses the proposed method, including image category information extraction and the integrated sampling strategy. Section 4 describes the accuracy of the proposed algorithm for obtaining image category information and the reliability of the proposed method through experiments. Finally, in Section 5, we present conclusions and outline future work.

## 2. Related work

Uncertainty Sampling is grounded in the core principle of selecting samples for which the model exhibits the highest uncertainty, aiming to minimize annotation costs while maximizing model performance. This approach has expanded from traditional classification tasks to regression problems, achieving widespread adoption in domains such as molecular dynamics simulations, object detection, and image analysis. However, the primary bottlenecks of this technology lie in its high parameter sensitivity, alongside the need to optimize computational costs and generalizability for broader applicability.

To minimize the additional workload of annotating training datasets and training networks in the active learning process for object detection tasks, our study focuses on improving uncertainty sampling strategies. The following introduces three classic uncertainty sampling strategies.

### 2.1 Least confidence sampling

The user sets the minimum confidence threshold, and samples with predicted confidence below this threshold will be selected, as specified in Formula 1:


$$\begin{array}{c} {x^{*}}_{LC}=\mathrm{\backslash arg}\max_{x}\left(1-P_{\theta }(\hat{y}|x)\right)=\mathrm{\backslash arg}\min_{x}\;P_{\theta }(\hat{y}|x) \end{array}$$ (1)

^y=\argmaxyPθ(y|x) denotes a trained machine-learning model with a parameter set θ. $\hat{y}$ represents the category predicted with the highest probability by the model for the category x.

Least confidence sampling is sensitive to model calibration errors. If the model’s confidence estimation is inaccurate (e.g., overconfidence), the sampling efficacy will degrade significantly.

### 2.2 Margin sampling

Margin sampling involves selecting samples where the difference between the model’s predictions for the maximum and second-maximum probabilities is minimal, as specified in Formula 2:


$${x^{*}}_{M}=\mathrm{\backslash arg}\min_{x}\left(P_{\theta }({\hat{y}}_{1}|x)-P_{\theta }({\hat{y}}_{2}|x)\right)\mathrm{\backslash ]}$$ (2)

${\hat{y}}_{1}$ and ${\hat{y}}_{2}$ respectively represent the category predicted as the most likely and the second most likely by the model for category x.

Margin sampling excels in binary classification tasks, but it exhibits poor adaptability to multiclass scenarios, neglects structural information in sample distributions, and may fail to accurately reflect the distance between samples and the true decision boundary for neural network softmax outputs.

### 2.3 Image entropy sampling

In mathematics, entropy can be used to measure the uncertainty of a system. Larger entropy indicates greater uncertainty, while smaller entropy indicates lower uncertainty. Therefore, in binary or multiclass scenarios, one can choose samples with relatively high entropy as potential annotation data, as specified in Formula 3:


$${x^{*}}_{H}=\mathrm{\backslash arg}\max_{x}\left(-\sum {\mathrm{\backslash nolimits}}_{i}P_{\theta }({\hat{y}}_{i}|x)⬝\ln P_{\theta }({\hat{y}}_{i}|x)\right)\mathrm{\backslash ]}$$ (3)

$P_{\theta }({\hat{y}}_{i}|x)$ represents the probability of the *i*th information state, and the value of entropy is calculated by multiplying the probability of each information state by its logarithm and then summing up and taking the negative value.

Entropy sampling calculations depend on probability distributions. In cases of imbalanced data categories or limited sample sizes (e.g., long-tailed distributions), traditional entropy metrics may overestimate or underestimate uncertainty, leading to sampling bias. The term “long-tail effect” describes a data distribution characterized by a substantial number of infrequent or rare instances which, despite their low individual occurrence, collectively form a significant portion of the dataset.

## 3. Category-enhanced uncertainty sampling

**Fig 1:**
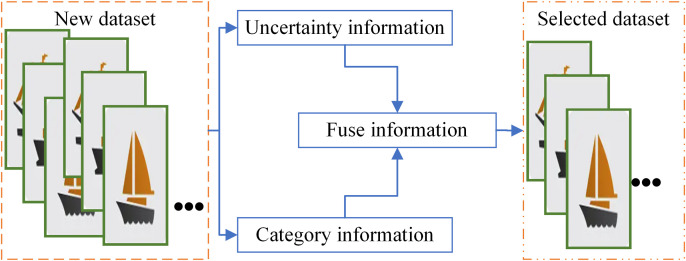
Active learning with integrated category information.

The fundamental principles of the proposed active learning algorithm based on unstable sampling and category information fusion are illustrated in Fig 1. The algorithm comprises three main components: obtaining category information for unlabeled novel samples, evaluating the uncertainty of unlabeled novel samples, and integrating the uncertainty with category information for a comprehensive evaluation of unlabeled novel samples. The final sampling strategy is determined using a comprehensive evaluation function, guiding the selection of a subset of samples from the unlabeled novel sample pool as the annotation set to achieve the goal of active learning.

The category information extraction method proposed in this study requires no training; it solely utilizes a publicly available pre-trained VGG16 to extract high-level features from images. The category features are then integrated effectively into traditional active learning methods based on unstable sampling, facilitating the extraction of valuable target data from large datasets.

### 3.1 Category feature extraction

**Fig 2:**
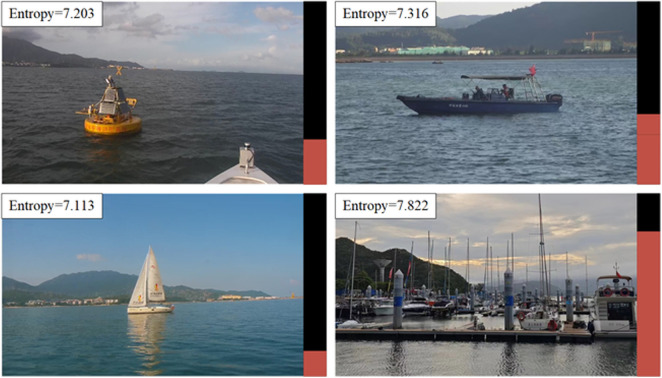
Entropy values of different categories of targets.

Classical uncertainty sampling strategies, due to the inability to obtain category information of images, rely solely on the complexity of the images as the sampling criterion. In the maritime domain of interest, common targets include buoys, various types of vessels, lighthouses, and docks. If considering only the inherent complexity of the images, dock category targets will always have a higher complexity than buoys. As shown in Fig 2, the entropy value for docks is the highest, followed by vessels, buoys, and lighthouses in descending order. This result aligns with human visual expectations. When there is a significant number of samples in the dock category, docks inevitably dominate the dataset after active learning sampling, compressing the occurrence frequency of other categories in the dataset.

**Fig 3:**
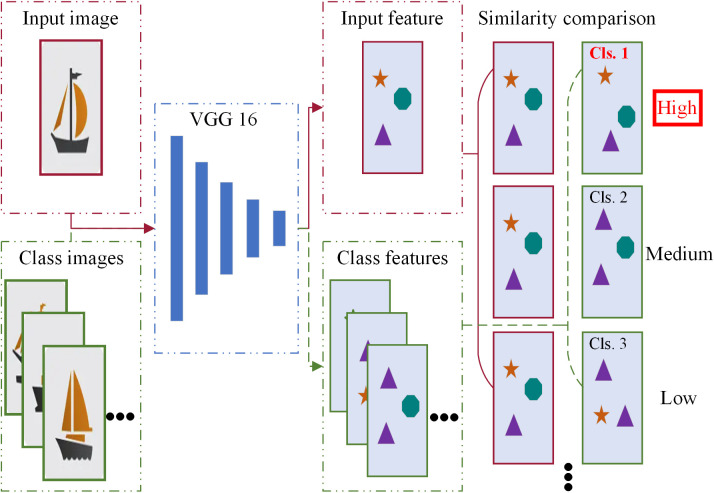
Workflow for extracting image category information.

In the process of deep learning, when there is a significant imbalance in the quantity of data between different categories in the dataset, it can significantly impact the final model’s performance. This paper proposes an efficient and accurate method for obtaining image category information without the need to train a deep learning model. The specific process is illustrated in Fig 3.

#### 3.1.1 Feature extraction.

First, a manually selected set of typical images from each class is assembled to form the labeled calibration set $C\_s\tan dard=\left\{x_{1},x_{2},...,x_{n}\right\}$ within the unlabeled dataset. Then, a pre-trained Convolutional Neural Network (CNN), such as the VGG16 or ResNet [21], is chosen. In this study, the pre-trained VGG16 from the torchvision library is employed.

Each image $X_{i}$ from the unlabeled dataset is individually passed through the pre-trained VGG16 alongside each known-class image $x_{j}$ from the labeled calibration set. This process allows the extraction of the output from the last layer of the VGG16, capturing the high-level features of the images.

#### 3.1.2 Feature similarity analysis.

Input the unclassified image $X_{i}$ and the known classified image $x_{j}$ into the VGG16, as shown in Equation 4:


y=VGG16(x) (4)

Obtaining the corresponding high-level features $\left(Y_{i},y_{j}\right)$, where $Y_{i}$ represents the high-level features of the image $X_{i}$ to be classified, and $y_{j}$ represents the high-level features of the known-class image $x_{j}$. The cosine similarity $s_{ij}$ between features $Y_{i}$ and features $y_{j}$ is computed to determine whether the two images belong to the same category, as shown in Equation 5:


$$s_{ij}=\cos\left(\theta \right)=\frac{Y_{i}\cdot y_{j}}{\left\|Y_{i}\right\|\left\|y_{j}\right\|}$$ (5)

Finally, the category of the unlabeled image $X_{i}$ is determined based on the category represented by the image $x_{j}$ with the highest cosine similarity, as shown in Equation 6:


$$C_{i}=\max\left(s_{i1},s_{i2},...,s_{ij},...,s_{in}\right)$$ (6)

### 3.2 Integrated sampling framework

After obtaining the category distribution of the unlabeled image set, the sampling strategy can be determined by integrating image instability and category information. When the category distribution of the dataset is relatively balanced, the sampling strategy should ensure a relatively balanced distribution in the final sampled dataset. In this case, the sampling mainly relies on image instability within each category dataset. When the category distribution of the dataset is imbalanced, priority should be given to sampling small-sample datasets. Lower category weights should be applied to datasets with higher proportions, and within the same category dataset, the sampling is primarily based on image instability, as shown in Formula 7:


$$P_{i}=\lambda_{c}\frac{\sum_{j=1}^{n}N_{c_{j}}}{N_{c_{i}}}+\lambda_{u}x^{*},x^{*}\in \left({x_{L\text{C}}}^{*},{x_{M}}^{*},{x_{H}}^{*}\right)$$ (7)

$N_{C_{i}}$ represents the quantity of the current image belonging to the category i, $\lambda_{c}$ represents the weight of category information in the sampling strategy, and $\lambda_{u}$ represents the weight of instability information in the sampling strategy. By setting the values of $\lambda_{c}$ and $\lambda_{u}$, the emphasis of the sampling strategy can be altered. When λc/λcλu\nulldelimiterspaceλu is relatively large, there is a tendency to sample data from rarer categories. When λc/λcλu\nulldelimiterspaceλu is relatively small, there is a tendency to sample data with more complex image information. $x^{*}$ represents any one of the three classical uncertainty sampling strategies.

## 4. Experimental evaluation

### 4.1 Experimental setup

#### 4.1.1 Experimental dataset.

To validate the effectiveness of our proposed active learning method, experiments were conducted on two tasks: object detection and image classification.

**Fig 4:**
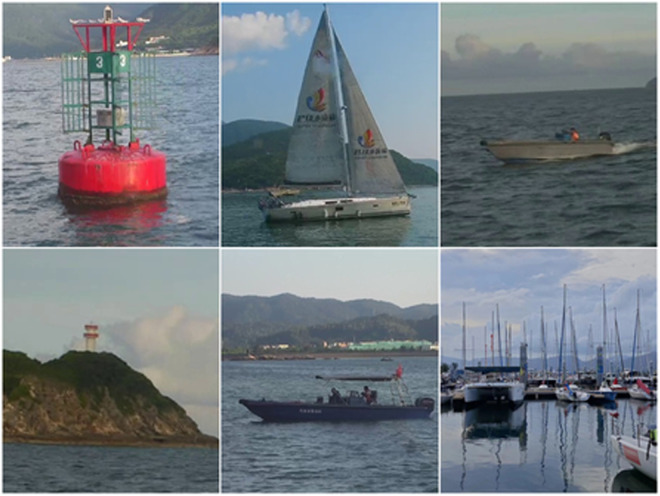
Taihu ship dataset.

For the object detection task, three datasets were employed: MS-COCO [22], VOC2007 [23], and the Taihu Ship Dataset. The Taihu Ship Dataset, constructed by the China Ship Scientific Research Center, comprises 6,000 images across three scenarios: docks, lakes, and ocean environments, as shown in Fig 4. It encompasses 10 classes of objects, including buoys, various types of vessels, and lighthouses, and has been publicly released in relevant artificial intelligence competitions. The MS-COCO dataset is widely used for multi-label image analysis, containing 82,081 training samples and 40,504 validation samples across 80 categories, with an average of 2.9 labels per image. VOC 2007 is a classical multi-label image dataset consisting of 9,963 images spanning 20 category labels.

For the image classification task, we utilized the CIFAR dataset [24], which encompasses two distinct tasks: a coarse-grained classification task with 10 classes and a fine-grained classification task with 100 classes, totaling 30,000 samples.

The dataset settings for this experiment are presented in Table 1:

**Table 1:** The dataset settings.

Dataset Name	Data Categories	Training Set	Test Set
Taihu	Self-built Dataset, 10 categories	4500 images	1500 images
VOC 2007	Public Dataset, 20 categories	6800 images	2200 images
MS-COCO	Public Dataset, 20 categories	10000 images	3000 images
CIFAR-10	Public Dataset, 10 categories	10000 images	3000 images
CIFAR-100	Public Dataset, 100 categories	10000 images	3000 images

#### 4.1.2 Active learning comparative algorithms.

In this experiment, the comparative algorithms include the following five:

1) Lowest Confidence Sampling (LC);
2) Image Entropy Sampling (IE);
3) Edge Sampling (EDGE);
4) Bayesian Active Learning by Disagreement (BALD [25]);
5) A Core-set Approach (CSA [26]);
6) Active Learning Algorithm Based on Deep Learning (GMM);
7) Active Learning Method Based on Unstable Sampling with Fused Category Information (Our).

#### 4.1.3 Algorithms used in the experiment.

The experiment will be conducted on two tasks: object detection and image classification. The typical YOLOv5 [27] is selected for the object detection task, and ResNet is selected for the image classification task.

#### 4.1.4 Experimental environment.

The experimental environment for this study is presented in Table 2.

**Table 2:** The experimental environment.

Parameter	Detail
CPU	Inter® Core™ i9-13900K
GPU	1*GeForce RTX 4090
Development language	Python 3.9
Integrated environment	Pycharm 2019
OS	Windows 10
Deep learning framework	Pytorch 1.13.1
CUDA version	11.6

### 4.2 Category extraction performance

**Fig 5:**
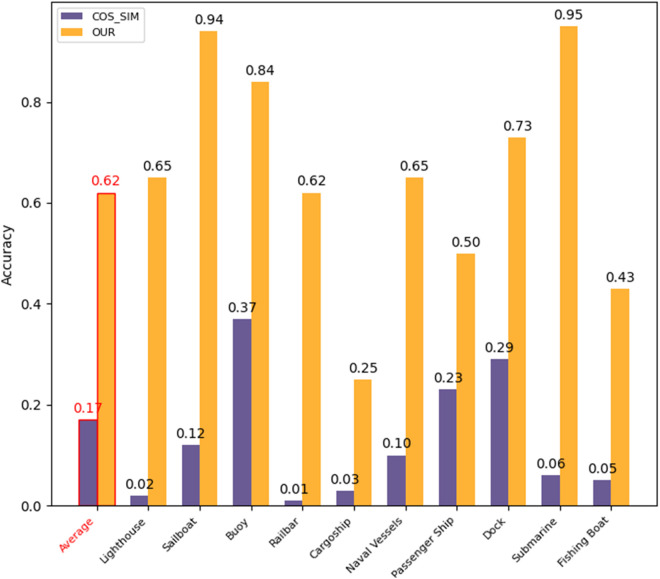
Classification accuracy.

An efficient image classification algorithm, combining the pre-trained VGG16 with cosine similarity, is compared with the ablation experiment results using only the cosine similarity classification algorithm, as shown in Fig 5. The average classification accuracy of the efficient image classification algorithm reaches 62%, significantly higher than the 17% achieved by the ablated algorithm. Especially in certain categories, such as “sailboat” and “submarine,” the classification accuracy is as high as 95%. This experimental result was obtained by considering only a single sample as the reference for each category in the sample set. Poor results may occur when the sample features fail to fully cover the sample set of that category.

For categories like “cargo ship” and “passenger ship,” there are often distinctions between large and small cargo ships, as well as large and small passenger ships. Using a single sample to represent the entire sample set can impact the algorithm’s classification ability. The solution is simple: in categories with poor classification performance, manually selecting multiple samples as references can significantly improve classification accuracy.

### 4.3 Results and discussion

Currently, in research related to active learning algorithms, there is no clear metric to assess the effectiveness of the algorithms. Therefore, this experiment will validate through three aspects:

a) Analyze whether the category distribution of datasets sampled by various algorithms is balanced. If the algorithm can extract more data from small samples and the overall category distribution of the sampled subset is close to the source dataset, the algorithm performs better.
b) Analyze the computational efficiency of various algorithms. Since the purpose of active learning algorithms is to save researchers’ time, algorithms with less time consumption during the dataset sampling process perform better.
c) Analyze how the data sampled by active learning algorithms have a positive impact on object detection and image classification tasks, and evaluate which active learning algorithm has better performance by comparing the mAP index of object detection and the accuracy index of image classification separately.

#### 4.3.1 Dataset category distribution.

**Fig 6:**
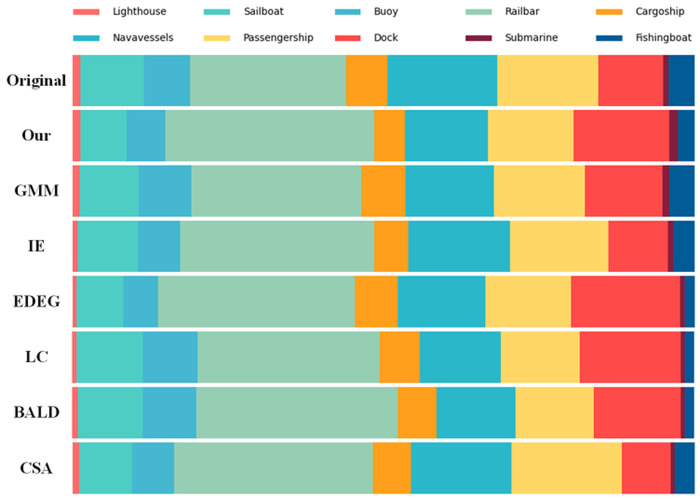
Category distribution of sampled datasets.

Fig 6 illustrates the category distribution of datasets sampled using five different active learning methods. LC, EDGE, IE, BALD and CAS can select data with rich image content, but due to the lack of attention to dataset category information, this may result in an excessive amount of complex image data (e.g., “dock” type) and insufficient data for some small samples (e.g., “lighthouse” type, “Submarine” type). In contrast, our algorithm and the GMM algorithm can sample complex images while prioritizing the selection of small-data categories, ensuring dataset reliability and balance. Due to the higher classification accuracy of deep learning algorithms, the GMM algorithm exhibits greater precision in filtering small-sample data.

#### 4.3.2 Computational performance.

**Fig 7:**
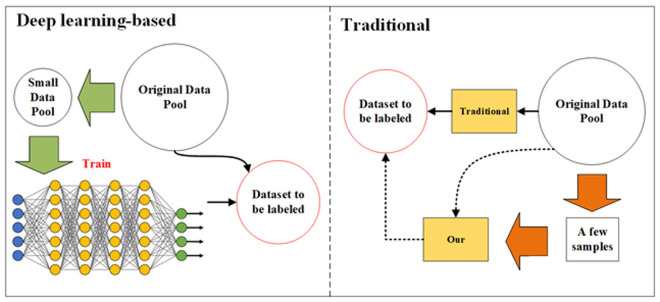
Analysis of pre-sampling overhead in different active learning methods.

As shown in Fig 7, traditional active learning methods that use instability sampling (e.g., LC, EDGE, IE, and BALD) can begin sampling immediately without any dataset preprocessing, resulting in zero initialization time. In contrast, deep learning-based active learning approaches like GMM require pretraining on a subset of labeled data to enable feature extraction before active learning can commence. While our algorithm’s computational efficiency is comparable to IE, it introduces some overhead due to the requirement for expert-guided selection of representative samples from the target dataset.

BALD can be computationally intensive, particularly when applied to large-scale problems, due to its requirements for posterior sampling or maintaining multiple models. CSA offers better computational efficiency compared to BALD by working with a reduced dataset, though the core-set construction process itself may still require significant computational resources depending on the chosen methodology.

As shown in Table 3, traditional active learning methods maintain consistently low computational overhead throughout their execution, requiring only CPU resources. Our algorithm achieves similar computational efficiency while leveraging GPU acceleration for inference tasks. In contrast, deep learning-based methods like GMM demonstrate significantly higher computational demands – approximately five times that of traditional approaches – and require GPU resources for both training and inference phases.

**Table 3:** The time consumption of algorithms (Datasets: Taihu).

Algorithms	The time consumption before sampling (h)	The time consumption of sampling (h)	Total time consumption of sampling (h)
LC	0	0.4	0.4
EDGE	0	0.7	0.7
IE	0	1.3	1.3
BALD	0	3.9	3.9
CSA	0.5	1.0	1.5
GMM	7.3	3.8	11.1
Our	0.2	1.7	1.9

To validate the versatility and generalizability of the category information extraction component, it was integrated into the LC and EDGE frameworks. Experimental results demonstrated that, while the original performance of LC and EDGE incurred an average time overhead increase of 0.5 hours, the long-tail effect in the ampled datasets was significantly reduced, and their balance was also improved.

#### 4.3.3 Detection and classification performance.

A) The object detection task

**Fig 8:**
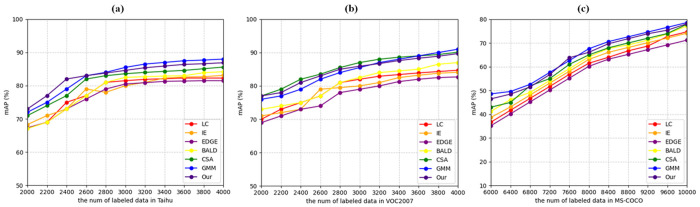
Results of active learning on object detection. (a) Taihu dataset. (b) VOC 2007 dataset. (c) MS-COCO dataset.

Different active learning algorithms are employed to sample subsets with varying data sizes in three datasets. YOLOv5 are trained using these datasets, and their performance is illustrated in Fig 8.

The results on the Taihu and VOC2007 datasets (Fig 8A and Fig 8B) demonstrate strong performance of our proposed method in object detection tasks. On the Taihu dataset, our approach achieves competitive mAP scores, particularly in the early stages (2000–2600 labeled samples) where it shows comparable or occasionally better performance than GMM, while consistently outperforming traditional methods like LC, IE, and BALD. For VOC2007, our method maintains stable performance growth as the number of labeled samples increases from 2000 to 4000, achieving final mAP scores above 85%. Although GMM shows marginally better results in some cases, our method demonstrates robust performance across different data volumes and maintains a clear advantage over other baseline approaches, particularly in the middle stages of the active learning process (2600–3400 samples).

The experiments on the more challenging MS-COCO dataset (Fig 8C) further validate the effectiveness of our approach when dealing with complex, real-world scenarios. Starting from a baseline of around 35% mAP with 6000 labeled samples, our method shows consistent improvement as more data is added, eventually reaching approximately 70% mAP with 10000 labeled samples. The performance curve closely tracks that of GMM, demonstrating comparable learning efficiency, especially in the range of 7200–8800 labeled samples. A notable strength of our method is its stable and consistent performance improvement across all three datasets, suggesting robust generalization capability across different object detection scenarios. While maintaining competitive performance with GMM, our approach shows particular advantages in its reliable sample selection strategy, evidenced by the smooth learning curves and consistent performance gains across different dataset scales.

B) The image classification task

**Fig 9:**
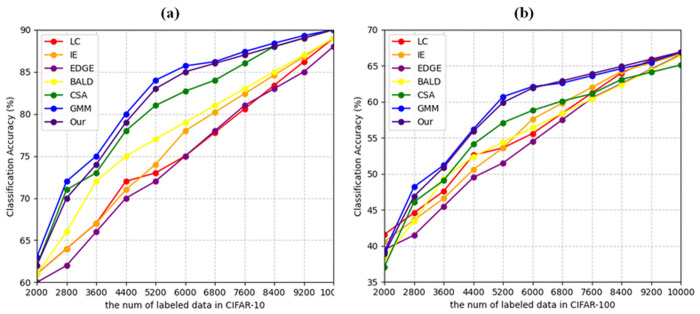
Results of active learning on image classification. (a) CIFAR-10 dataset. (b) CIFAR-100 dataset.

Different active learning algorithms are employed to sample subsets with varying data sizes in two datasets. ResNet are trained using these datasets, and their performance is illustrated in Fig 9.

The experimental results on CIFAR-10 (Fig 9A) demonstrate the effectiveness of the proposed method across different sizes of labeled data. Our method shows competitive performance, achieving accuracy comparable to the state-of-the-art GMM approach and consistently outperforming several baseline methods including LC, IE, EDGE, BALD, and CSA. Specifically, when the number of labeled samples is between 4400 and 7600, our method maintains stable performance with classification accuracy ranging from 75% to 85%, showing robust learning capability. The performance gap between our method and traditional approaches like LC and IE becomes more pronounced in this range, indicating better sample selection efficiency.

On the more challenging CIFAR-100 dataset (Fig 9B), our method exhibits similar trends while dealing with increased classification complexity. The results indicate that our approach maintains competitive performance compared to GMM, with only marginal differences in accuracy across different labeled data sizes. A notable strength of our method is its consistent performance improvement as the number of labeled samples increases from 2000 to 10000, achieving final accuracy around 65%. This steady improvement suggests that our method effectively identifies and selects informative samples even in scenarios with many classes. While GMM shows slightly better performance in some cases, our method still demonstrates robust learning capability and maintains advantages over other baseline methods, particularly in the latter stages of the active learning process (beyond 6000 labeled samples) (Fig 9).

The deep neural network model utilized by GMM possesses a stronger feature extraction capability, particularly when dealing with high-dimensional and complex data such as images. It can construct more abstract and discriminative representations, which enables GMM to more effectively identify informative boundary samples during the sample selection process. In contrast, our method, which does not rely on retraining deep features, may exhibit relatively weaker capability in capturing the underlying class structure.

Additionally, GMM performs full model retraining or fine-tuning at each iteration of the active learning cycle, allowing it to quickly adapt to newly labeled samples. In our approach, to improve sampling efficiency and reduce computational overhead, the classifier is not fully updated in each round. This design choice may lead to suboptimal decision boundary adjustments, especially during early stages or in tasks with significant distributional shifts, thereby affecting classification performance.

## 5. Conclusions

This paper introduces a category-enhanced active learning method designed to address the limited annotated data in the maritime domain, a challenge for large-scale deep learning applications. Our approach improves upon traditional instability sampling by integrating category information, enabling efficient selection of high-value data without requiring deep neural network training for each new dataset. Compared to recent active learning models reliant on initial annotated data, this method is more time- and resource-efficient, with better scalability across diverse application scenarios. Currently, category extraction requires expert input. Future work will aim to automate this process by developing intelligent category selection algorithms using unsupervised or semi-supervised techniques, alongside adaptive weighting mechanisms for real-time feedback adjustments. This would enhance sampling efficiency and extend our model’s utility to real-time applications in maritime surveillance and autonomous navigation.
